# Proteomic Analysis of *Bt cry1Ac* Transgenic Oilseed Rape (*Brassica napus* L.)

**DOI:** 10.3390/plants12122319

**Published:** 2023-06-15

**Authors:** Zheng-Jun Guan, Min Zheng, Zhi-Xi Tang, Wei Wei, C. Neal Stewart

**Affiliations:** 1 Department of Life Sciences, Yuncheng University, Yuncheng 044000, China; zhengjunguan@126.com; 2 State Key Laboratory of Vegetation and Climate Change, Institute of Botany, Chinese Academy of Sciences, Beijing 100093, China; zhengmin0816@126.com (M.Z.); tangzhixi@ibcas.ac.cn (Z.-X.T.); 3 Department of Hotel Management, Linyi Technician Institute, Linyi 276005, China; 4 Department of Plant Sciences and Center for Agricultural Synthetic Biology, University of Tennessee, 2505 EJ Chapman Drive, Knoxville, TN 37996-4561, USA; nealstewart@utk.edu

**Keywords:** *cry1Ac* gene, oilseed rape, proteomics, 2D gel electrophoresis

## Abstract

Oilseed rape (*Brassica napus* L.) is an important cash crop, but transgenic oilseed rape has not been grown on a commercial scale in China. It is necessary to analyze the characteristics of transgenic oilseed rape before commercial cultivation. In our study, differential expression of total protein from the leaves in two transgenic lines of oilseed rape expressing foreign Bt Cry1Ac insecticidal toxin and their non-transgenic parent plant was analyzed using a proteomic approach. Only shared changes in both of the two transgenic lines were calculated. Fourteen differential protein spots were analyzed and identified, namely, eleven upregulated expressed protein spots and three downregulated protein spots. These proteins are involved in photosynthesis, transporter function, metabolism, protein synthesis, and cell growth and differentiation. The changes of these protein spots in transgenic oilseed rape may be attributable to the insertion of the foreign transgenes. However, the transgenic manipulation might not necessarily cause significant change in proteomes of the oilseed rape.

## 1. Introduction

With the rapid development of genetic engineering technology, the application and effects of transgenic plants have gradually attracted public attention worldwide [1,2]. Transgenic technology can produce novel foods more effectively and reduce the need for pesticides, leading to less environmental pollution [3,4]. In particular, Bt toxins isolated from *Bacillus thuringiensis* have been expressed in transgenic plants to confer inherent pest resistance. Bt crops have been overwhelmingly successful and beneficial with respect to increasing yields and reducing chemical pesticide use [5]. However, the technology might have unintended negative impacts on the environment and human health. Nutrition compounds in transgenic Bt crops may be changed, and may even generate toxic compounds due to of the unknown effects of exogenous genes [6,7,8,9,10,11]. Therefore, Bt plants are commonly selected as experimental systems in studies that focus on detecting potential effects of exogenous genes in transgenic plants.

Oilseed rape (*Brassica napus* L.) is an important oil and cash crop, and it is a major source of edible vegetable oil and proteins. As such, the improvement of rape quality and yield has received increased attention. Herbicide-resistant transgenic oilseed rape was one of the earliest biotech crops developed and has a large range of applications. At present, herbicide-tolerant transgenic oilseed rape has been commercialized in the United States, Canada, Australia and other countries [12]. Although insect-resistant Bt oilseed rape has not been commercialized, it has served as a model biotech crop for studying the effects of transgenic plants (e.g., Cao et al. 2014; Liu et al. 2018) [13,14]. Owing to current methods of transgene insertion, transgenic plants may exhibit random genomic side effects such as variable gene expression, mutations to endogenous loci at the points of insertion, homologous gene expression inhibition effects (i.e., silencing), activation effects and other unanticipated changes in crop phenotypes. Therefore, it is necessary to assess potential unexpected consequences of transgenic Bt oilseed rape before commercial cultivation.

Due to the potential unknown effects and unpredictability of exogenous genes, proteomic technology is an effective and direct method for detecting the unintended effects of transgenic plants [15,16]. Differential proteomics is a major research area that mainly aims to detect and confirm different proteins produced by special stimulation between two or more groups of samples [17,18,19]. Two-dimensional gel electrophoresis, a core technique of differential proteomic analysis, can isolate different proteins that exist in plants [20]. Previous studies have determined proteome changes in transgenic plants by using proteomics [21,22,23,24,25,26]. Up to now, the research on proteomic analysis of transgenic plants has not raised any new safety issues [25,26]. In our study, in order to better understand the influencing mechanism of the exogenous *Bt cry1Ac* gene on oilseed rape, a proteomic approach was used to analyze the differential expression of proteins in transgenic Bt oilseed rape.

## 2. Results

### 2.1. Comparative Proteomic Analysis of Transgenic and Non-Transgenic Bt Oilseed Rape Leaves

**Figure 1:**
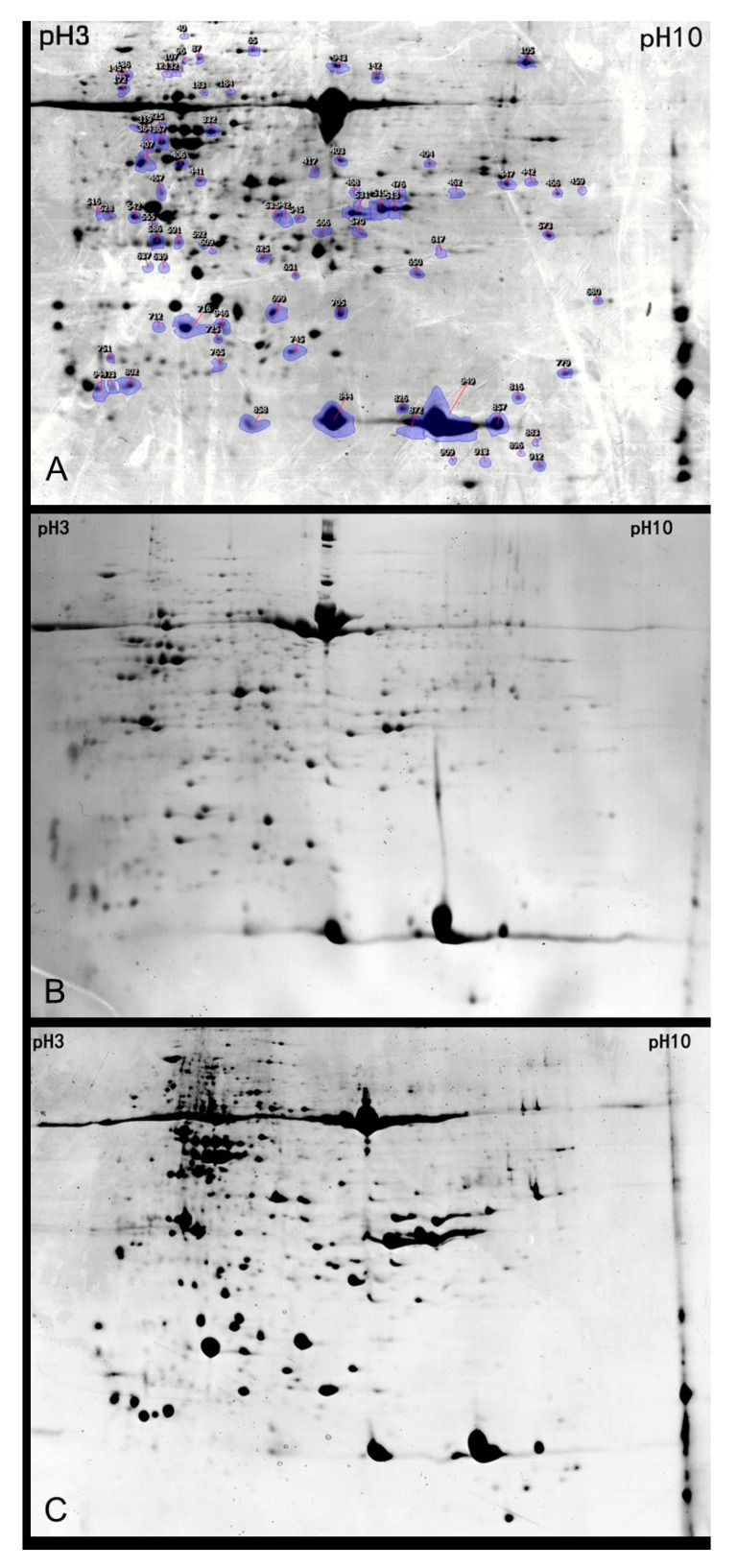
Two-dimensional gel electrophoresis of non-transgenic Westar (**A**), transgenic GT1 (**B**), and GT9 (**C**) oilseed rape. Potentially differential protein spots were identified and marked in blue for further analysis (A).

To evaluate the unintended effects of a foreign gene on the leaf expression profile of oilseed rape, differential proteomics between transgenic oilseed rape lines (GT1 and GT9) and the control plant (‘Westar’) were analyzed. Two-dimensional (2D) gel electrophoresis patterns of leaf proteins with high resolution and reproducibility were successfully obtained. After staining with Coomassie brilliant blue, results for 2D gel electrophoresis of total proteins from the leaves of two lines of transgenic oilseed rape and the non-transgenic control were obtained (Figure 1). Protein spots were detected in the leaves of transgenic oilseed rape, and the number and score of protein spots that matched between transgenic oilseed rape and its control were counted (Table 1). 30 differentially expressed protein spots were successfully detected and identified in the 2 transgenic lines, including 21 upregulated expressed protein spots and 9 downregulated protein spots. Protein spots that were changed in both transgenic lines were further analyzed.

### 2.2. Identification and Functional Evaluation of the Differentially Expressed Proteins

The detected spots of differentially expressed proteins were excised from the 2D gels and identified using MALDI-TOF/TOF and database searches (such as NCBInr and KEGG). Fourteen differentially expressed protein spots were successfully identified, including eleven upregulated expressed protein spots and three downregulated protein spots. Table 1 provides information such as the Mascot score, NCBI accession ID and name and molecular weight of the successfully identified proteins.

To evaluate the characteristics of the identified proteins, the theoretical and experimental ratios of molecular weight (MW) and isoelectric point (pI) were determined, respectively (Table 1). The closer the theoretical and experimental values of the identified proteins are, the greater the certainty that the identification made by means of Mass Spectrometry (MS) database searching will be the MS identification obtained. About 80% of the theoretical and experimental molecular weight values of the identified proteins were similar, but their pI values were different, indicating that the identified proteins had different characteristics and possible isoforms of the MS data.

**Figure 2:**
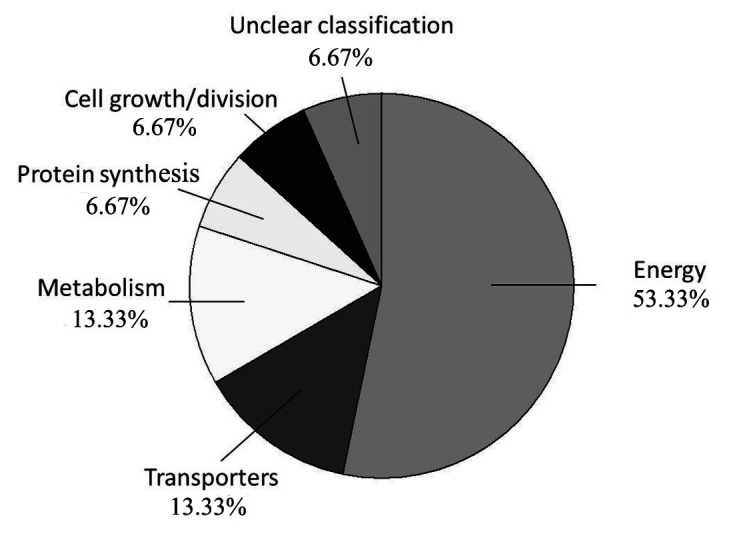
Graphic representation of the functional distribution of differentially abundant protein species (%) identified in non-transgenic Westar and transgenic GT1 and GT9.

The identified proteins were classified into six categories on the basis of their biological activities (Table 1 and Figure 2). In this study, 53.33% (eight spots) of the identified proteins were related to energy functions, 13.33% (two spots) to transporters, 13.33% (two spots) to metabolism, 6.67% (one spot) to protein synthesis, 6.67% (one spot) to cell growth/division, and 6.67% (one spot) presented an unclear classification.

In this study, several differentially expressed proteins—including ribulose-1,5-bisphosphate carboxylase/oxygenase large subunit, transketolase, ribulose bisphosphate carboxylase small chain 1B, ribulose bisphosphate carboxylase/oxygenase activase, ribulose bisphosphate carboxylase small chain F1 and ribulose bisphosphate carboxylase large chain—were found to be upregulated and involved in the energy metabolism pathway. Furthermore, some proteins that showed downregulated expression, such as calcium-transporting ATPase and V-type proton ATPase catalytic subunit A, may be related to transporters (Table 1).

## 3. Discussion

To investigate and characterize the different proteins between transgenic and non-transgenic oilseed rape, proteomic detecting tools (2DE and MS) were applied to identify the differentially expressed proteins between two transgenic Bt oileed rape lines (GT1 and GT9) and its non-transgenic parent plant (Westar) to evaluate proteomic changes between transgenic and non-transgenic plants in our study. Only the protein spots that changed in both transgenic lines in comparison to the non-transgenic plants were further analyzed in order to detect changes that were likely caused by the insertion of foreign transgenes and genetic manipulation. Fourteen protein spots were detected and identified, although the difference seems to not be biologically significant, with a ratio ranging from 0.85 to 1.12. The results suggested that transgenic manipulation might not cause differences in *B. napus* plant proteomes, while such a change could be considered significant when comparing a single transgenic line to its parent plant, e.g., Liu et al. [23]. In this study, these detected proteins of slight difference were mainly related to energy functions, indicating that proteins involved in energy were likely affected by the transgenic manipulation. Yang et al. [27] investigated different proteins between transgenic and non-transgenic rice plants (*Oryza sativa* L.) via comparative proteomic analysis, and a similar result was obtained, that the most abundant category was energy-related proteins among those identified. Liu et al. [23] employed proteomic approaches to study protein abundance changes in seeds from the Bt transgenic line GT1 of oilseed rape (*B. napus*), and eight proteins were more abundant in transgenic oilseed rape seeds than in non-transgenic seeds. The current work studied the proteomic change in the leaf of oilseed rape caused by gene transformation, and no protein with the previous study was detected on seed proteomes. It was suggested that those detected proteins in seeds [23] and in leaves (current study) could be tissue-specific, and the impact of inserted transgenes could be also different between seeds and leaves.

In this study, the highest proportion of detected proteins was related to photosynthesis. Further works are needed to elucidate the potential impact on plant productivity. Ribulose-1,5-bisphosphate carboxylase/oxygenase (rubisco), which is the most abundant protein in plants, is widely found in organelles with photosynthetic functions. Rubisco, composed of one larger and one smaller subunit, is a key enzyme that affects the carbon assimilation rate during photosynthesis. Unlike other enzymes, rubisco is a bifunctional enzyme that catalyzes carboxylation and oxygenation, an intersection of recycling reactions of both photosynthetic carbon reduction and oxidation [28]. In our study, seven upregulated protein spots were related to rubisco subunits. In particular, upregulated ribulose bisphosphate carboxylase/oxygenase activase (protein spot 367) in the leaf of transgenic oilseed rape, which was identified in this study, may change the conformation of the rubisco active site, helping to accelerate the carboxylation process and avoid digestion [29,30]. The expression levels of rubisco in both transgenic GT1 and GT9 were higher than in the non-transgenic parent plant, likely because insertion of the Bt gene required numerous rubisco molecules in order to express the inserted transgene in oilseed rape. The increased abundance of rubisco is likely able to help plants adapt to their environment by conferring a higher carbon assimilation rate [31]. This could be a plant strategy to deal with potential adverse impacts caused by transgene insertion. The co-expressed rubisco gene in three transgenic rice lines was remarkably upregulated under salt stress [32]. It implicated that rubsico could be susceptive to transgene manipulation and environmental growth stress.

Transketolase plays a key role in the Calvin cycle of photosynthesis and is involved in the synthesis of nucleic acids, carbohydrates, amino acids and lipids. Transketolase is identified as a target of herbicidal substance α-terthienyl, revealed by a proteomics study [33]. Overexpression of the transketolase gene promotes chilling tolerance by increasing the activities of photosynthetic enzymes, alleviating oxidative damage and stabilizing cell structure in *Cucumis sativus* L. [34]. Transgenic *Chlamydomonas reinhardtii* cells that overexpressed transketolase of *Pyropia haitanensis* grew better than wild-type cells in response to osmotic stress [35]. Compared to wild-type cotton, five upregulated transketolase protein spots were identified in a transgenic cotton line with a *crylAc* gene from *Bacillus thuringiensis* (BT) [36]. In our study, it can be inferred that the increase of transketolase in transgenic oilseed rape is likely caused by Bt gene insertion.

V-type proton ATPase catalytic subunit A (V-H+-ATPase), a kind of H^+^-ATPase, is mainly responsible for catalyzing the hydrolyzation of ATP. This enzyme plays a key role in ion balance within plant cells, and it may modulate the stress resistance of plants (including salt, drought, cold and excessive heavy metal stresses) [37,38]. For example, salt stress reduced the V-H^+^-ATPase and the V-H^+^-PPase activity in potato cultivars [39]. Calcium-transporting ATPase is a Ca^2+^ transportation system, and it is crucial in regulating intracellular or extracellular Ca^2+^ concentration and signal transduction [40]. V-H^+^-ATPase may be related to calcium-transporting ATPase [41]. The results showed that the expression of V-H^+^-ATPase has the same decreasing tendency as that of calcium-transporting ATPase in transgenic oilseed rape (GT1 or GT9). Therefore, it could be assumed that despite the insect-resistant features, decreased expression of the two enzymes may weaken the abiotic stress resistance of transgenic plants. To test this hypothesis, future experiments could be conducted to assess the performance of the transgenic plant under abiotic stress conditions.

Alanine aminotransferase 2-like belongs to the pyridoxal phosphate multigene family, and it may help in regulating carbon and nitrogen metabolism in plant cells [42]. A decrease in the expression of alanine aminotransferase 2-like in transgenic oilseed rape (GT1 or GT9) may impact the relevant physiological metabolism and reduce resistance to anti-anoxia and pathogens. Glutamine synthetase (GS) is an important enzyme involved in the assimilation of inorganic nitrogen into organic forms in higher plants [43]. Salt stress induced glutamine synthetase activity in the roots and the leaves of *Trigonella foenum-graecum* L. plants. [44]. GS2-cosuppressed rice plants exhibited a poor plant growth phenotype and a poor nitrogen transport ability [45]. Six genes encoding GS-protein were found and identified from the transcriptome data of the asparagus (*Gracilaria lemaneiformis* L.) genome [46]. In this study, increased expression of GS in transgenic oilseed rape (GT1 or GT9) could enhance the regulation of nitrogen metabolism and improve salt tolerance in transgenic plants.

Ribosomal protein L11 is a highly conserved protein located at the base of the L7/L12 stalk of the ribosome, and is mainly involved in promoting ribosomal RNA folding during protein synthesis [47]. Expression of ribosomal protein L11 in transgenic oilseed rape (GT1 and GT9) increased, likely due to the synthesis of the Bt protein, which requires the participation of a large number of ribosomes. Cis-zeatin-O- glucosyltransferase may stimulate the activity of cytokinins in plants, thereby regulating a series of physiological and biochemical processes (e.g., stimulating growth, retarding senescence and plant stress resistance) [48]. Upregulated expression of cis-zeatin-O- glucosyltransferase in the leaves of transgenic oilseed rape could delay plant senescence to a certain degree and improve the production of transgenic oilseed rape.

In this study, owing to the insertion of the Bt gene, changes in the transgenic oilseed rape leaf proteome were detected, and these changes played important roles in processes such as energy conversion, protein transport and metabolism. These results provided useful information for further illuminating the potential effects of transgenic oilseed rape on human health and environment. Our study inferred that exogenous DNA in a host oilseed rape genome might affect plant photosynthesis, which requires further study. Incidental differences among differences in transgenic-line-associated, photosynthesis-related proteins may have effects on other plant traits, such as biomass production and chlorophyll concentrations, even though no differences were observed here (data not shown). Although there were some unintended protein variations in transgenic oilseed rape leaves, there were no obvious functional proteomic changes produced in the oilseed rape leaf proteome. This study presented a well-established relationship between the identified proteins in transgenic oilseed rape via MS/MS and the databases. The role of each identified protein was curated. The proteins identified in this study were not unique to oilseed rape, but are common among plants. Hence, the identified differences are deemed to not be novel or hazardous, and no significant change in proteomes was found. As such, these transgenic oilseed rape lines likely have no non-target effects in the proteomes. Further works should focus on the safety issues that were solely caused by the expression of Bt Cry1Ac toxin.

In summary, the differential expression of total protein in transgenic oilseed rape was compared using a proteomic approach. Eleven upregulated expressed protein spots and three downregulated protein spots were analyzed and identified. Those protein spots in oilseed rape leaves that related to energy conversion, protein transport, and metabolism may be affected by the transgenic procedure. The results showed that some unintended protein variations, due to the transformation of foreign transgenes in oilseed rape leaves, may not be biologically significant. However, further confirmation of the safety implications of the changes must be considered in risk assessment, especially under environmental change conditions.

## 4. Materials and Methods

### 4.1. Plant Materials and Planting

The following three plant types were used in this study: *Brassica napus* ‘Westar’ (non-transgenic maternal parent) and GT1 and GT9 (two transgenic *B. napus lines*). *B. napus* Westar, a spring-type oilseed rape, was transformed with genetically linked GFP and the Bt (Cry1Ac) gene regulated by independent CaMV 35S promoters in the pSAM12 plasmid [49], and two of the GFP/Bt transformed lines (GT1 and GT9) were used in this current study.

Greenhouse experiments of three biological replicates were conducted at the Institute of Botany, Chinese Academy of Sciences, Beijing, China. The three types of plants were grown in plastic basins in the greenhouse. The potting mix consisted of vermiculite, peat moss and clay soil in the proportion 1:1:1 (*v*:*v*). The plants were cultured for 16 h of supplemental light per day at a temperature ranging from 18 to 25 °C. The leaves in four to five-leaf stages were selected as experimental materials.

### 4.2. Protein Extraction and Quantification

Proteins were extracted from the top expanded leaves of the two transgenic lines and the non-transgenic *B. napus* using the method described by Joosen et al. [50]. The presence of transgenes was confirmed by PCR with specific primers (Cry1Ac transgene: 5′-ATTTGGGG-AATCTTTGGTCC-3′ and 5′-ACAGTACGGATT-GGGTAGCG-3′; GFP gene: 5′-TACCCAGATCATATGAAGCGG-3′ and 5′-TTGGGATCTTTCGAAA GGG-3′) for Bt and GFP transgenes at the 4–5 leaf stage at 4 weeks after seed germination [49]. After 1 g of the leaf was ground up in a mortar with liquid nitrogen, proteins were extracted using 10 mL of extraction buffer (10% *w*:*v* trichloroacetic acid/acetone, 0.07% *w:v* DTT), depolymerized using lysis buffer (7 M urea, 2 M thiourea, 4% *w:v* CHAPS, 1 mM PMSF, 50 mM DTT, 0.5% *w*:*v* Triton X-100, and 0.5% *v*:*v* IPG-buffer) and subsequently measured using the Protein Quantification Kit by Bradford method (Beijing Boling Kewei Bio-Technique Co., LTD, Beijing) using BSA (- Sigma-Aldrich. Inc., Beijing) as the standard [51].

### 4.3. 2D Gel Electrophoresis

The 10 mg protein sample was mixed with 200 µL of isoelectric focusing buffer (7 M urea, 2 M thiourea, 4% *w*:*v* CHAPS, 50 mM DTT, 0.5% *v*:*v* IPG-buffer and 0.001% *w:v* bromophenol blue) and loaded onto 18 cm IPG linear dry strips (pH 3–10). After passive rehydration for 14 h, the strips were focused using the Protean Isoelectric Focusing System (Bio-Rad), and the following program was used: 2 h at 50 V, 1 h at 100 V, 1 h at 200 V, 1 h at 500 V, 1 h at 1000 V, 5 h of a linear gradient to 8000 V and 5 h at 8000 V [50].

The strips were equilibrated at room temperature for 15 min in the equilibration solution (6 M urea, 0.375 M Tris-HCl [pH 8.8], glycerol, 2% SDS with 1% DTT, and 0.001% bromophenol blue, followed by carboxymethylation with 2.5% iodoacetamide). The equilibrated strips were run on 12.5% SDS polyacrylamide gels at 5 mA/gel for 45 min and 20 mA/gel for 4–6 h until the dye front reached the bottom of the gel. Proteins were visualized using Coomassie Brilliant Blue G-250 staining after 1 h of protein fixation in a solution containing 40% ethanol and 10% acetic acid. Destaining was performed with the same fixing solution for 1 h, followed by 5 washes with water.

### 4.4. Image and MS Analysis

Well-separated gels of the three independent biological replicates were used for proteomic comparisons. The gels were scanned with the UMAX Power Looker 2100XL Scanner (Shiqun International Trading Co., Ltd., Shanghai, China) and analyzed for proteome differences. Progenesis Samespots DIGE enable (v4.5) visual tools (CloudScientific Technology Co., Ltd., Shanghai, China) were used for image analysis, spot detection, matching between gels and normalization. Three biological repeats for each sample were examined, and the results were shown in average ± SD (*n* = 3). Spots of interest were manually excised from the Gel Analysis Program (GAP) stained 2DE gels.

Protein spots with significant changes were analyzed using matrix-assisted laser desorption/ionization-time of flight mass spectrometry (MALDI-TOF-MS) [52]. Spots were considered reproducible if they were detected in all the biological replicates. Protein spots were considered to be differentially accumulated when the change was more than 1.5-fold with statistically significant differences (*p* < 0.05) [23].

Protein identification was performed by searching for MS and MS/MS data in the National Center for Biotechnology Information (NCBI) databases using a built-in Mascot server (V2.1, Matrix Science, London, UK). Proteins were identified using a minimum of two MS/MS spectra matching the databank sequence. All identifications were manually validated. The search parameters were used as follows: trypsin was selected as the digestive enzyme, carbamidomethylation of cysteine as a fixed modification, oxidation of methionine as a variable modification, 100 ppm mass tolerance for precursor ions, 0.2 Da of peptide and fragment mass tolerance and one missed cleavage. The proteins for which the Mascot scores were more than threshold score 55 were considered to be reliably identified (*p* < 0.05).

## Figures and Tables

**Table 1:** Distribution of protein spots in groups and their MS identification between two transgenic oilseed rape lines (GT1 and GT9) and the non-transgenic oilseed rape plants (Westar).

Spot Number ^a^	Protein Name	Accession No. ^b^	Score ^c^	Theor. pI ^d^	Exp. pI ^d^	Theor. MW ^e^	Exp. MW ^e^	Ratio ^f^
Energy								
802 (↑)	Ribulose-1,5-bisphosphate carboxylase/oxygenase large subunit	gi|11466371	86	5.06	6.14	52,405	53,186.5	1.07
65 (↑)	Transketolase	gi|18411711	97	5.81	5.94	79,837	80,374.4	1.06
858 (↑)	Ribulose bisphosphate carboxylase small chain 1B	gi|15240912	68	6.27/	7.59	20,155	20,558.2	1.06
367 (↑)	Ribulose bisphosphate carboxylase/oxygenase activase	gi|297612474	82	4.98	7.56	38,775	39,108.5	1.08
699 (↑)	Ribulose-1,5-bisphosphate carboxylase/oxygenase large subunit	gi|11466371	134	5.06	6.14	52,405	53,186.5	1.12
844 (↑)	Ribulose bisphosphate carboxylase small chain F1	gi|132091	390	6.27	8.23	14,358	20,455.2	1.06
476 (↑)	Ribulose bisphosphate carboxylase large chain	gi|2500677	78	6.28	6.39	48,953	49,069.4	1.08
872 (↑)	Ribulose bisphosphate carboxylase small chain F1	gi|132091	103	6.27	8.23	14,358	20,455.2	1.06
Transporters								
586 (↓)	Calcium-transporting ATPase	gi|302756809	63	8.07	6	112,406	113,321.0	0.93
87 (↓)	V-type proton ATPase catalytic subunit A	gi|15219234	96	4.53	5.11	68,682	69,111.0	0.93
Metabolism								
651 (↓)	Alanine aminotransferase 2-like	gi|30698866	59	4.95	5.95	59,380	59,986.2	0.85
325 (↑)	Glutamine synthetase	gi|12643761	114	4.24	6.16	47,214	47,714.0	1.09
Protein synthesis								
513 (↑)	Ribosomal protein L11	gi|56404772	66	9.60	9.27	14,973	15,151.0	1.10
Cell growth/division								
319 (↑)	Cis-zeatin O-glucosyltransferase	gi|242093988	63	4.79	6.06	50,372	50,813.9	1.05

a: Assigned spot numbers as indicated in Figure 1; b: Accession numbers according to NCBInr; c: The Mascot searched score (M. S.) against the database NCBInr; d: The experimental and theoretical pI of the identified proteins; e: The experimental and theoretical mass (Da) of the identified proteins; f: The normalized spot volume in GT1/GT9 leaves divided by the normalized volume in Westar leaves. The upwards arrow ‘↑’ and the downwards arrow ‘↓’ stands for upregulated and downregulated protein spot, respectively.

## Data Availability

The data presented in this study are available on request from the corresponding author.
